# Activation of Electrophile/Nucleophile Pair by a Nucleophilic and Electrophilic Solvation in a S_N_Ar Reaction

**DOI:** 10.3389/fchem.2018.00509

**Published:** 2018-10-23

**Authors:** Bruno Sánchez, Cristian Calderón, Ricardo A. Tapia, Renato Contreras, Paola R. Campodónico

**Affiliations:** ^1^Departamento de Química, Facultad de Ciencias, Universidad de Chile, Santiago, Chile; ^2^Centro de Química Médica, Facultad de Medicina, Clínica Alemana Universidad del Desarrollo, Santiago, Chile; ^3^Facultad de Quimica, Pontificia Universidad Católica de Chile, Santiago, Chile

**Keywords:** solvent effects, ionic liquids, catalysis, anion effect, preferential solvation

## Abstract

Nucleophilic aromatic substitution reactions of 4-chloroquinazoline toward aniline and hydrazine were used as a model system to experimentally show that a substrate bearing heteroatoms on the aromatic ring as substituent is able to establish intramolecular hydrogen bond which may be activated by the reaction media and/or the nature of the nucleophile.

**Graphical Abstract 1:**
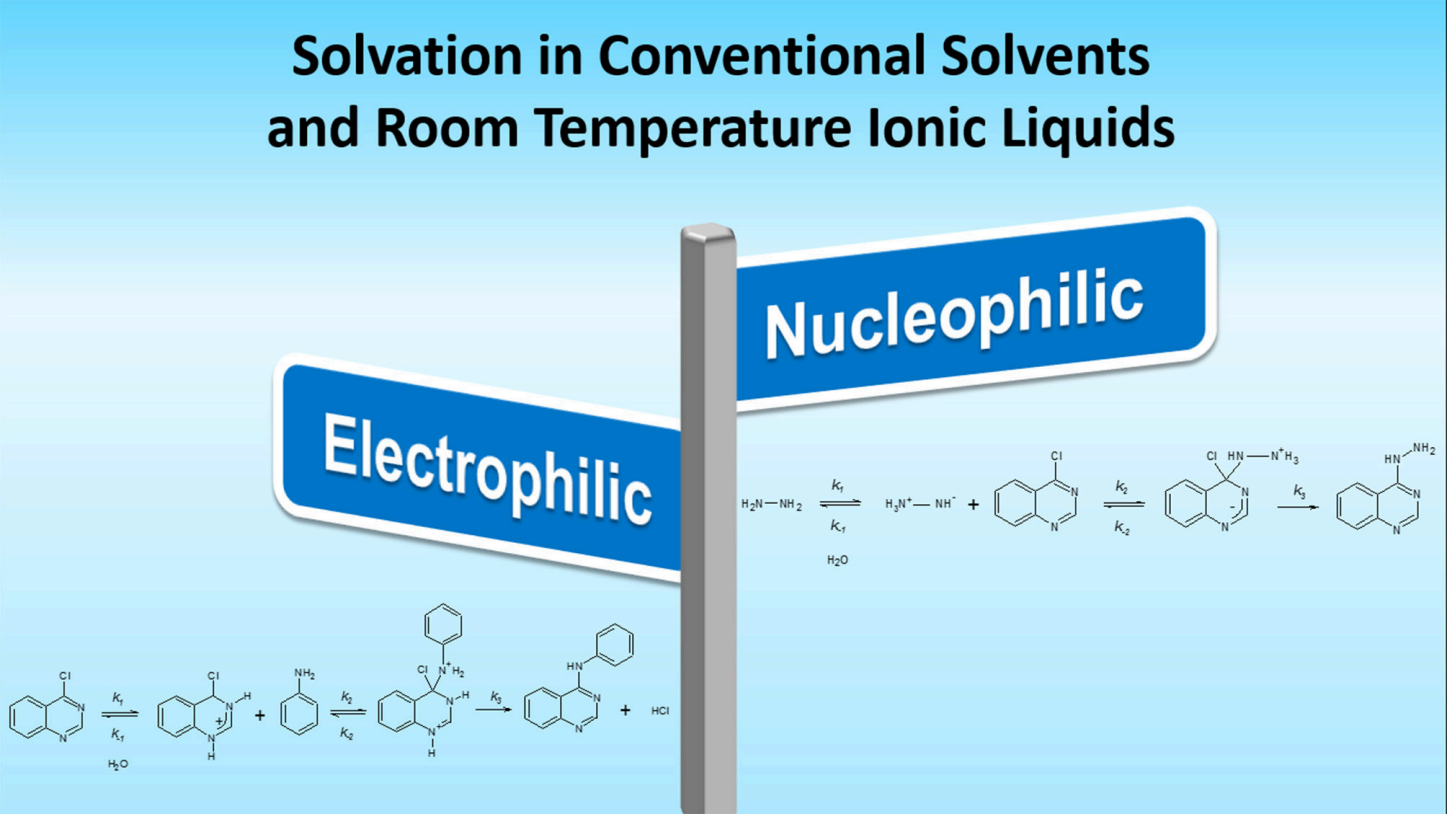
**Graphical Abstract**.

## Introduction

The electrophilicity (ω^+^) and nucleophilicity (ω^−^) concepts (Ingold, 1929, 1933, 1934) are related to electron-deficient (electrophile) and electron-rich (nucleophile) species (Jaramillo et al., 2006). These concepts were early introduced by Ingold in 1934 and they are based on the valence electron theory of Lewis (Lewis, 1923) and the general acid-base theory of Brönsted and Lowry (Brönsted, 1923; Lowry, 1923; Cedillo et al., 2007).

The solvent effect at a microscopic scale could be studied considering the possible interactions using Lewis concepts. The solvent effects usually are studied in aromatic nucleophilic substitution (S_N_Ar) reactions, because the reaction media involves the stabilization of species associated to the Potential Energy Surface (PES) such as: electrophile/nucleophile (*E*^+^/*Nu*^−^) pair, transition state (TS) and intermediate structures, respectively (Ormazábal-Toledo et al., 2013c; Gazitúa et al., 2014; Alarcón-Espósito et al., 2015, 2016, 2017). Specific interactions such as hydrogen bond (HB), π-π and p–π stacking are expected to shed insights on the responses of these species in the bulk and specific *E*^+^/*Nu*^−^ pair-solvent interactions would determine selectivity, reaction rates and mechanisms (Ormazábal-Toledo et al., 2013c; Gazitúa et al., 2014; Alarcón-Espósito et al., 2015, 2016, 2017; Marullo et al., 2016).

Considering that the solvation and catalysis phenomena are mainly determined by HB interactions, would be more appropriate to use the Lewis acidity/basicity than Brönsted acidity/basicity concepts in order to perform scales. The main reason is because the Brönsted acidity/basicity scale is refers to different chemical processes where the acidic/basic hydrogen atom is completely transferred or accepted in the form of a proton, while the Lewis acidity/basicity scale is fundamentally a regional property and not a global one (Ormazábal-Toledo et al., 2013c; Gazitúa et al., 2014; Alarcón-Espósito et al., 2015, 2016, 2017; Marullo et al., 2016). Solvent effects can be split into two types: non-specific and specific interactions, including all the possible interactions that can occur between solvent and the *E*^+^/*Nu*^−^ pair (Chiappe et al., 2011). Preferential solvation (Mancini et al., 1999; Ormazábal-Toledo et al., 2013c; Alarcón-Espósito et al., 2015) may be defined into the form of specific *E*^+^/*Nu*^−^ pair-solvent interactions that describes local solvation, defined as a “first solvation shell.” Local solvation may be classified as “electrophilic” or “nucleophilic” solvation (Winstein et al., 1951; Olah and Klumpp, 2004; Ormazábal-Toledo et al., 2013c).

Electrophilic solvation represents the specific interaction through an HB with the hydrogen atom of the solvent, whereas nucleophilic solvation describes a specific interaction through a HB between an acidic hydrogen atom of the *E*^+^/*Nu*^−^ pair and the solvent (Ormazábal-Toledo et al., 2013c). The effect of the HB in S_N_Ar was early studied by Bernasconi et al. (Bunnett et al., 1955; Bernasconi and De Rossi, 1976) and recently by our group (Ormazábal-Toledo et al., 2013a,b,c; Gallardo-Fuentes et al., 2014; Alarcón-Espósito et al., 2015; Contreras et al., 2015) from experimental and theoretical point of view in order to explain the observed reactivity trends (Parr et al., 1999; Contreras et al., 2003; Ormazábal-Toledo et al., 2013a).

**Scheme 1:**
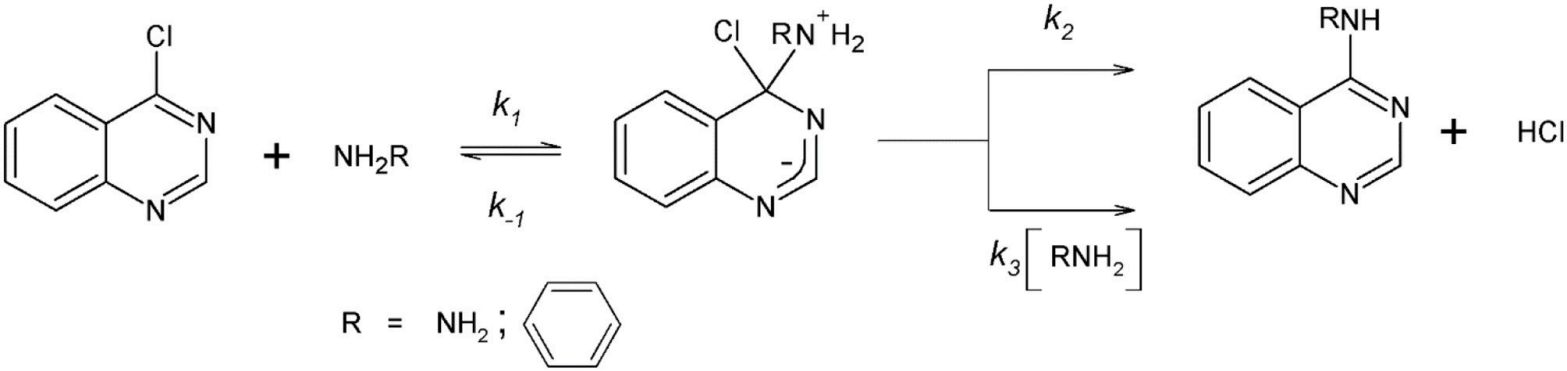
General reaction mechanism for the S_N_Ar of 4-chloroquinazoline and the amines studied.

Scheme 1 shows the S_N_Ar process considered in this work. Note that, the first step on the reaction pathway corresponds to the formation of a zwitterionic complex named Meisenheimer complex (MC) from which two processes for its decomposition have been postulated:(Banjoko and Babatunde, 2004; Um et al., 2007; Ormazábal-Toledo et al., 2013a,b,c Terrier, 2013; Gallardo-Fuentes et al., 2014; Gazitúa et al., 2014; Alarcón-Espósito et al., 2015, 2016, 2017; Contreras et al., 2015) (a) expulsion of the leaving group (LG) followed by fast proton loss to give the reaction product (*k*_2_ in Scheme 1), and (b) the base-catalyzed deprotonation of the MC that loss the LG to give the reaction product (*k*_3_ in Scheme 1) (Alarcón-Espósito et al., 2017).

Solvent effects studies in S_N_Ar have been addressed experimentally in order to get detailed information about phenomena that are not observable and occurring at microscopic levels (Ormazábal-Toledo et al., 2013b,c; Alarcón-Espósito et al., 2015; Contreras et al., 2015). The model systems are the reactions between the substrate or electrophile named 4-chloroquinazoline and two amines of different nature (aniline and hydrazine), respectively (see Scheme 1).

The main goal of this work is to analyze the activation of the *E*^+^/*Nu*^−^ pair by nucleophilic or electrophilic solvation given by conventional solvents like water and organic solvent and ionic liquids (IL). This article will be focused on the discussion of the solvent on the reactivity of the *E*^+^/*Nu*^−^ pair based on a detailed kinetic study and its incidence on the reaction mechanisms.

ILs at room temperature (RTIL) are defined as molten salts (composed entirely of cations and anions) that melt below 100°C (Welton, 1999; Crowhurst et al., 2004; Weingärtner, 2008) with remarkable physicochemical properties (Freemantle, 1998). The high combinatorial flexibility has converted these materials into “design solvents” or “task specific” solvents (Welton, 1999; Weingärtner, 2008; Gazitúa et al., 2014) whose properties can be specified to suite the requirements of a particular reaction (Reichardt and Welton, 2011; Gazitúa et al., 2014; Alarcón-Espósito et al., 2016). Details of structures and acronyms of the IL used in this study are in Scheme 1. RTILs have been classified as Lewis acid/base with the ability of the anion to accept a HB and to donate an HB in the case of the cation forming the IL (Crowhurst et al., 2003; Ab Rani et al., 2011; Contreras et al., 2013; Gazitúa et al., 2015).

## Materials and methods

### Reagents and solvents

All reagents and solvents used were the commercially available from Sigma-Aldrich and Merck. The certificate of analysis guarantees purity ≥ 99%.

### Purity of the ionic liquids

The series of ionic liquids used were purchased from Merck and used as delivered. The specifications are as follows: purity (HPLC) > 98%; identity (NMR) passed test; halides (IC) < 0.1%; water (KF) < 0.1%.

### Kinetic measurements

The kinetic of the reactions in aqueous media were measured by a diode array spectrophotometer HP 8453 at 25°C at an ionic strength 0.2 M in KCl. The kinetic measurements in conventional organic solvents and ionic liquids were made in the absence of KCl. The formation of a reaction product was monitored at 330 nm. The substrate concentration was 1.09 × 10^−4^ M. Under excess of nucleophile, pseudo-first-order rate coefficients *k*_*obs*_ were obtained. The reactions were carried out at three pH values (pH = *pK*_*a*_ and pH = *pK*_*a*_ ± 0.3) thereby establishing an equilibrium between the free nucleophile and its protonated form. The total concentration of each nucleophile comes from free nucleophile and its protonated form (Gazitúa et al., 2014; Calfuman et al., 2017).

### Spectroscopic measurements

Polarity determinations were performed by monitoring the spectroscopic behavior of Reichardt's dye. A stock solution of the probe was prepared in ethanol, and aliquots of the respective dye solution was added to the studied RTILs, previously dried overnight (70°C, under vacuum), and the added volume of ethanol was removed by evaporation under vacuum, followed by treatment of the solutions with a nitrogen stream. Absorption spectra were recorded in an Agilent 8453 UV-Vis spectrometer using 1 cm path length quartz cells. Et30 was calculated from the longest wavelength absorption maxima of Reichardt's dye, according to Equation 1.

(1) $$Et_{30}=\frac{28591.5}{\lambda_{\max}}$$

C343 steady-state fluorescence measurements were performed using a Perkin Elmer LS-55 fluorescence spectrometer, using an excitation wavelength of 340 nm and 1 cm path length quartz cells. The final spectra considered for each measurement corresponds to the average between 20 collected spectra. The fluorescent probe was incorporated into the samples following the same procedure for Reichardt's dye (Sánchez et al., 2018).

### Product study

The presence of the kinetic reaction products: 4-anilinoquinazoline and 4-hydrazinoquinazoline (Asif, 2014), respectively were validated spectrophotometrically by comparison of the UV-vis spectra at the end of the reactions with those authentic samples under the same experimental conditions (Alarcón-Espósito et al., 2017).

### Synthesis of 4-anilinoquinazoline

A solution of 4-chloroquinazoline (164.6 mg, 1.0 mmol) and aniline (279.4 mg, 3.0 mmol) in *n*-butanol (5.0 mL) was heated at reflux for 1.5 h. After the reaction mixture was concentrated under reduced pressure, the residue was purified by chromatography on silica gel using ethyl acetate: chloroform 2:1 to afford 4-anilinoquinazoline (155 mg, 70% yield, mp. 222–223°C (Lit. 222-224 °C) (Shen et al., 2011). ^1^H NMR (DMSO-*d*_6_): 9.80 (s, 1H), 8.59 (s, 1H), 8.56 (d, 1H, J = 8.0 Hz), 7.90-7.75 (m, 4H), 7.64 (t, 1H, J = 8.0 Hz), 7.40 (t, 2H, J = 8.2 Hz), 7.15 (t, 1H, J = 7.4 Hz). ^13^C NMR (DMSO-*d*_6_): 158.8, 154.4, 149.5, 139.1, 133.0, 128.4 (2C), 127.7, 126.2, 123.8, 122.9, 122.5 (2C), 115.1.

## Results and discussion

**Scheme 2:**
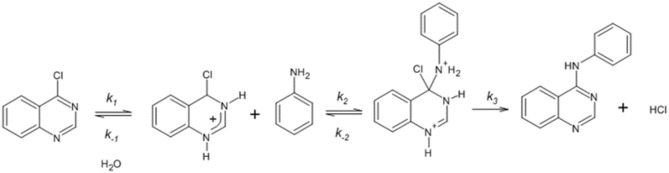
Proposed reaction mechanism for the reaction between 4-chloroquinazoline and aniline.

The studied reactions followed a stepwise mechanism, where the non-catalyzed route is the rate determining step (RDS). Under the experimental conditions, for the amines considered, single products (4-anilinoquinazoline and 4-hydrazine quinazoline, see Scheme 2), respectively were observed and monitored by UV-Vis spectrophotometry. The pseudo-first-order rate constant (*k*_*obs*_) for the reactions can be expressed as Equation 2, in which [Nu] represents the concentration of nucleophile (Alarcón-Espósito et al., 2017)

(2) $$\frac{k_{obs}}{\left[Nu\right]}=\frac{k_{1}(k_{2}+k_{3}[Nu])}{\left(k_{-1}+k_{2}+k_{3}\left[Nu\right]\right)}$$

The microconstants (*k*_1_, *k*_−1_ *and k*_3_, *respectively*) are associated to the reaction mechanism of an S_N_Ar reaction (see Scheme 1). Note that, the values of *k*_*obs*_ are in accordance with eq. 3 were *k*_0_ and *k*_*N*_ are the rate coefficients for solvolysis and nucleophilic attack, respectively. These values are obtained as the intercept (*k*_0_) and slope (*k*_*N*_) of linear plots of E3 (Choi et al., 2002; Castro et al., 2009).

(3) $$k_{obs}=k_{0}+k_{N}\left[Nu\right]$$

S_N_Ar reactions may present two mechanistic trends: *(i)* assuming that: *k*_2_ + *k*_3_[*Nu*] < < *k*_−1_, Equation 2 can be simplified to give Equation 4, where $K_{1}=\;\frac{k_{1}}{k_{-1}}$. Plots of *k*_*obs*_ *vs*. [*Nu*] show curves upward in accordance with Equation 4, thereby indicating that the reaction proceeds through a rate-limiting proton transfer mechanism (Banjoko and Babatunde, 2004; Um et al., 2007; Ormazábal-Toledo et al., 2013a,b,c; Terrier, 2013; Gallardo-Fuentes et al., 2014; Gazitúa et al., 2014; Alarcón-Espósito et al., 2015, 2016, 2017; Contreras et al., 2015).

(4) $$k_{obs}=K_{1}k_{2}\left[Nu\right]+K_{1}k_{3}{\left[Nu\right]}^{2}$$

*(ii)* when *k*_−1_+ *k*_2_ ≫ *k*_3_ the formation of the MC intermediate is the rate determining step, where *k*_*obs*_ is given by Equation 5: (Banjoko and Babatunde, 2004; Um et al., 2007; Ormazábal-Toledo et al., 2013a,b,c; Terrier, 2013; Gallardo-Fuentes et al., 2014; Gazitúa et al., 2014; Alarcón-Espósito et al., 2015, 2016, 2017; Contreras et al., 2015).

(5) $$k_{obs}=k_{N}\left[Nu\right]\;and\;k_{N}=\frac{k_{1}k_{2}}{k_{-1}+k_{2}}$$

Plots of *k*_*obs*_ *vs*. [*Nu*] show straight lines in accordance with Equation 5, thereby indicating that the reaction proceeds through a non-catalyzed mechanism (*k*_3_ *channel in* Scheme 1).

Table 1 shows the nucleophilic rate coefficients (*k*_*N*_) and *pK*_*a*_ values for both amines in aqueous media at 25°C and ionic strength 0.2 M in KCl. Note that, the values accompanying *k*_*N*_ coefficients correspond to the error associated to the slope. From Table 1 it is clear that the reaction is pH-dependent. The kinetic results for aniline suggest that *k*_*N*_ values are improved when the acidity of the media is increased. However, the inverse effect is observed with hydrazine. Note that, the nature and reactivity of the amines are different and their *pK*_*a*_ values were statistically corrected by using *p* (numbers of protons which can be deprotonated from the conjugate acid of the nucleophile) and *q* (numbers of nucleophilic sites of the nucleophile) (Bell, 1959; Um et al., 2007). Tables S1–S6 show the kinetic data in Supplementary Material.

**Table 1:** Nucleophilic rate coefficients and *pK*_*a*_ values for aniline and hydrazine in aqueous media at 25°C and ionic strength 0.2 M in KCl.

**Nucleophile**	***pK*_*a*_**	**pH**	**$k_{N}\;\text{(}s^{-1}M^{-1}\text{)}$**
Aniline	4.73	4.43	0.617 ± 0.016
		4.73	0.381 ± 0.012
		5.03	0.225 ± 0.013
Hydrazine	8.10	7.80	0.015 ± 0.001
		8.10	0.025 ± 0.003
		8.40	0.033 ± 0.002

**Figure 1:**
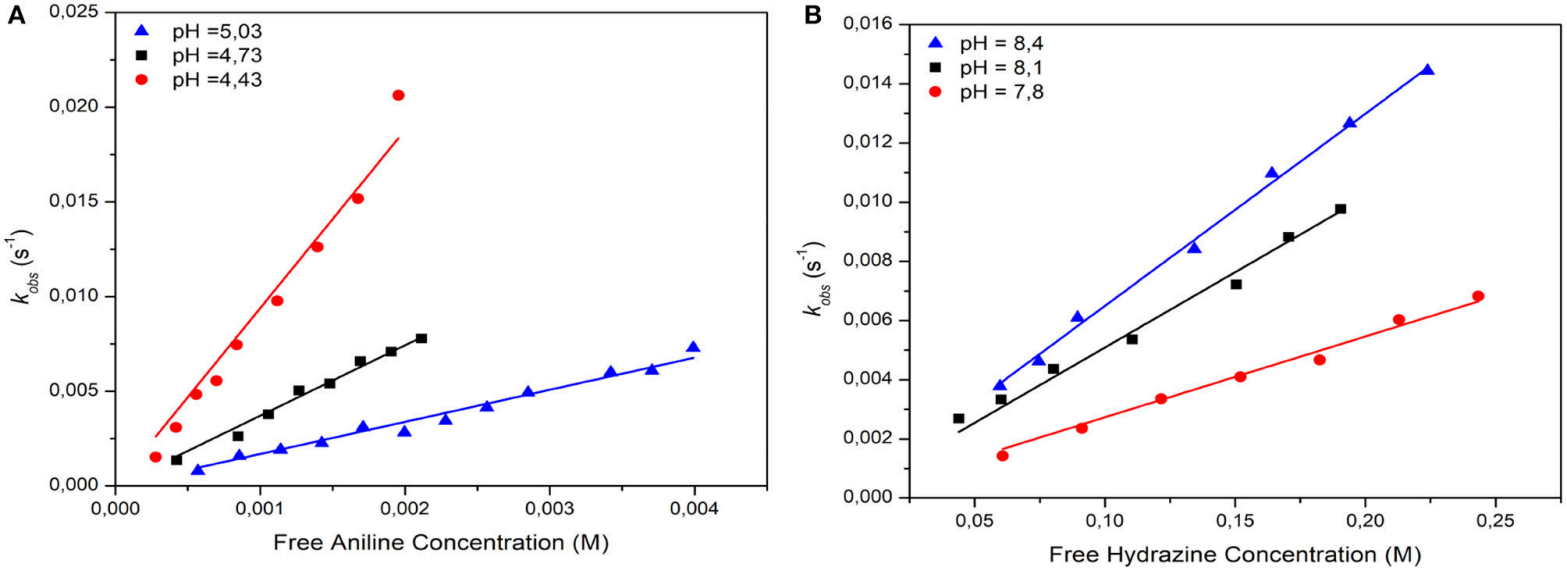
Plots of *k*_*obs*_ *vs*. free amine concentration in aqueous media **(A)** aniline and **(B)** hydrazine.

Figure 1 shows the plots of *k*_*obs*_ against free amine concentration expressed in Molar (M). Note that in Figure 1, the linear response for both amines in aqueous media discards a catalytic route exerted by a second molecule of nucleophile. Figure 1 shows that each linear response has an intercept close to the origin. This result would suggest that the step *(i)* can be safely discarded and the contribution of the reaction media may be irrelevant (Um et al., 2007).

**Scheme 3:**
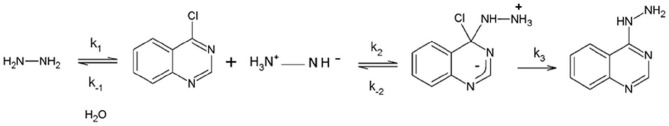
Proposed reaction mechanism for the reaction between 4-chloroquinazoline and hydrazine.

In summary, the kinetic data suggest that reactivity of the *E*^+^/*Nu*^−^pair is determined by an electrophilic & nucleophilic solvation, respectively. On other words, the HB that would be determining the reactivity of the studied reaction, could be established with the hydrogen atom of the solvent (electrophilic solvation) or between an acidic hydrogen atom of the *E*^+^/*Nu*^−^ pair and the solvent (nucleophilic solvation) (Ormazábal-Toledo et al., 2013c). See Scheme 3.

The reaction between aniline and 4-chloroquinazoline in aqueous media (Figure 1A) suggests that the substrate, in more acidic conditions, is protonated (see Table S7 and Figure S1). This electrophilic solvation promotes its reactivity toward aniline making it more susceptible to the nucleophilic attack. In order to validate this hypothesis, the reaction was performed in aprotic conventional organic solvents as: formamide, acetonitrile and dichloromethane, respectively. However, the kinetic studies under the same experimental conditions showed that the reaction did not proceed after 5 h in those solvents.

Based on these kinetic results it is possible to postulate at first glance that prior to the nucleophilic attack, there is a protonation step oriented toward the electrophile. (Campodónico et al., 2010) This step will induce an increased electrophilicity on the substrate making it susceptible to the attack of a nucleophile of low nucleophilic strength. Taking into account this additional step on the reaction mechanism, kinetic experiments were carried out in some RTILs such as: BMIMSO, EMIMDCN, BMIMDCN, BMIMPF_6_, BMIMBF_4_, and BMIMNTF_2_. The observed response only was in 1-ethyl-3-methyl imidazolium methane sulfonate (BMIMSO). For the other studied RTILs, the reaction did not proceed after 5 h. The elapsed time was an arbitrary kinetic criterion considered in this study in order to discriminate when the reaction proceeds (or not) toward the formation of reaction products.

The relationship between *k*_*N*_ values were about 30 times slower than water (see Table 1, *k*_*N*_ value at pH = *pK*_*a*_). The obtained *k*_*N*_ value for BMIMSO is of 0.0128 ± 0.0009 (*s*^−1^*M*^−1^). More details are in Figure S2 and Table S8. This information suggests that the reaction would proceed better in a protic RTIL. Preliminary data showed that propyl ammonium nitrate (PAN) was 68 times more reactive than BMIMSO with a *k*_*N*_ value of 0.8740 ± 0.0359 (*s*^−1^*M*^−1^). See Table S9 and Figure S2. These results shown that the nature of the cation/anion pair of the RTILs is determinant in the reaction pathway for this reaction (Hallett and Welton, 2011). The cation moiety of PAN would be playing a significative influence over the studied reaction. This effect may be traced to its Lewis basicity and the insignificant effect of the anion over the course of this reaction. This statement is supported on the kinetic response of the studied reaction on the analyzed RTILs which adds to the “task specific” concept, where determined RTILs can be specified to suite the requirements of a particular reaction (Welton, 1999, Weingärtner, 2008; Gazitúa et al., 2014; Alarcón-Espósito et al., 2016). Then, the capacity to donate an HB by ammonium moiety of PAN toward the substrate would be emulating the electrophilic solvation toward the 4-chloroquinazoline suggested in aqueous media. Scheme 2 shows the proposed mechanism for this reaction. In it is suggested a first step (*K*_1_) that corresponds to the protonation of the electrophile leading to the formation of a positive intermediate (Banjoko and Babatunde, 2004). This intermediate will be enhancing the electrophilicity of the substrate thereby improving its reactivity toward aniline. Note that, this route does not follow the formation of the traditional MC intermediate typical in a S_N_Ar reaction. However, this positively charged intermediate will suggest that the nucleophilic attack of the aniline molecule (*K*_2_) toward an activated substrate will be the slow step of the reaction pathway with a fast LG departure (*k*_3_) and deprotonation of it. The suggested RDS associated to the nucleophilic attack is attributable to a poor nucleophile (aniline) and a good LG (chlorine atom) added to a first protonation step toward the electrophile. On the other hand, the mentioned deprotonation step is not considered here, because it is very fast in comparison with the others steps on the reaction pathway. Note that, based on the previous kinetic analysis (linear plots of *k*_*obs*_ *vs*. [*Nu*]) the catalyzed route was discarded (*k*_3_ in Scheme 1). A definitive answer about the mechanism would be obtained through the full exploration of the PES that could contribute to add evidence on the proposed pathway.

On the other hand, the kinetic analysis for the reaction between 4-chloroquinazoline and hydrazine showed that it is improved at high pH values in aqueous media (see Figure 1B in the text). Scheme 3 shows the proposed mechanism for this reaction. In this case, the nature of the amine changed in comparison to aniline. In Scheme 3 is suggested a first step (*K*_1_) corresponding to the nucleophilic solvation of hydrazine, where hydrazine, highly reactive in a second step, will act as dipole reacting with the electrophile (*K*_2_) although the formation of a MC with a fast LG departure (*k*_3_) and deprotonation of it. More details below in the analysis of this reaction.

From Table 1 and considering *k*_*N*_ values at pH = *pK*_*a*_ for both amines, the relationships between *k*_*N*_ values was about 15 times slower than aniline. This fact is not in agreement with the *pK*_*a*_ values and the kinetic results. Hydrazine is an alpha nucleophile (Anderson and Jencks, 1960; Ormazábal-Toledo et al., 2013b; Gallardo-Fuentes et al., 2014). This compound has a lone pair vicinal to the attacking nitrogen atom (Ormazábal-Toledo et al., 2013b). Therefore, it should be expected an enhanced nucleophilicity toward the substrate. This fact, suggests that the reaction media has an improved effect over the reaction. Water molecule is polar, with high possibilities to establish HB interactions. Then, water molecules would be affecting the nucleophilic character of hydrazine via HB formation. However, the reaction of 4-chloroquinazoline with hydrazine in aqueous media (see Table 1) shows a catalytic behavior (see Figure 1B) similar to that found between 4-chloroquinazoline with aniline (see Figure 1A), but in an opposite acid/base conditions. This fact implies that the reaction media will be able to capture or/and to donate an hydrogen atom from the hydrazine molecule suggesting that the nucleophile will be acting as dipole (Kirby et al., 2006). This nucleophilic solvation will be promoting the reactivity of the hydrazine toward 4-chloroquinazoline through a strong nucleophile. Remember that, the substrate in more basic condition will be not activated. Then, this S_N_Ar reaction will follow the formation of an anionic intermediate. The same reaction in conventional organic solvents shown *k*_*N*_ values higher than water (see Table 1, *k*_*N*_ value at pH = *pK*_*a*_). The studied solvents were: ethanol, butanol, dioxane and acetonitrile, respectively. Tables S10–S13 and Figure S3 are given in SM. Aprotic solvents show *k*_*N*_ values in the same range than water (see Table 1 for hydrazine). The obtained values were 0.027 ± 0.002 and 0.012 ± 0.002 (*s*^−1^*M*^−1^) for dioxane and acetonitrile, respectively. However, ethanol showed an increased value of *k*_*N*_ (0.071 ± 0.001 *s*^−1^*M*^−1^) in comparison to water at pH = *pK*_*a*_ (0.025 *s*^−1^*M*^−1^). This protic solvent would open the possibility to establish an HB with the hydrazine shifting the equilibria toward its zwitterionic form.

Finally, this reaction was performed in ILs showing that the nature of the anion improves the reactivity, being at least 10 times higher than water and conventional organic solvents (DÁnna et al., 2010). The *k*_*N*_ values obtained in 1-butyl-3-methylimidazolium dicyanamide (BMIMDCN) and 1-butyl-1-methylpyrrolidinium dicyanamide (BMPYRDCN) were of 0.239 ± 0.027 (*s*^−1^*M*^−1^) and 0.325 ± 0.039 (*s*^−1^*M*^−1^), respectively. More details are in Tables S14, S15 and Figure S4. Note that, these RTILs have the same anion moiety. The selection is based on the high polarizability of the dicyanamide anion and its size (Gazitúa et al., 2014; Alarcón-Espósito et al., 2016). The aforementioned led us to analyze the solvation effect in terms of the kinetics in conventional solvents and its relationships on the reaction pathway. These results show a key role of the HB acidity (Lewis acidity) and the minor effect of the cation in the course of the reaction. In this way, this reaction is promoted when the anion moiety is able to capture an HB from the hydrazine emulating the same behavior of the reaction showed in aqueous media, enhancing the “anion effect” over the reaction (Alarcón-Espósito et al., 2016; Sánchez et al., 2018). The hypothesis of the zwitterionic form for hydrazine could be complemented with the report of one of our previous works, which describes a site activation problem for a pyrimidine derivative and benzohydrazide series in aqueous media (Gallardo-Fuentes et al., 2014). Both analysis allow us to suggest for this reaction an intramolecular HB formation that would operate as a perturbation that will produce a dual response at the reaction centers by enhancing the electrophilicity of 4-chloroquinazoline and the nucleophilicity of the hydrazine molecule (Gallardo-Fuentes et al., 2014). Then, the substrate that bear heteroatoms on the aromatic ring as substituent and the zwitterionic nucleophile would be able to establish an intramolecular HB between the α hydrogen atom on the ^+^NH_3_- moiety of hydrazine molecule and the nitrogen atom of the quinazoline moiety; the rate determining step being nucleophilic attack. See more mechanistic details in Scheme 3.

**Figure 2:**
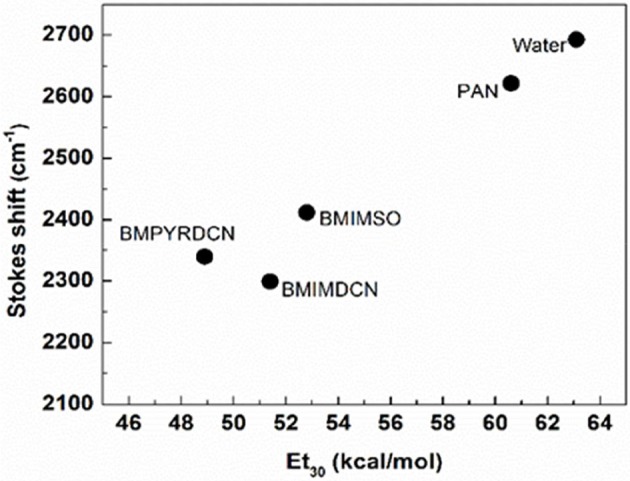
Plot of Coumarin 343 stokes shift vs. the Et_30_ values of the studied ionic liquids.

In order to further analyze the performance of the RTILs used in this study, steady state fluorescence studies were performed. Figure 2 shows the Stokes shift of the solvatochromic probe (coumarin 343, denoted C343) against to Et30 values for the used RTILs. Note that, water is considered as the reference solvent. C343 belongs to a family of 7-aminocoumarin derivatives (Kuznetsova and Kaliya, 1992) which has been used extensively as laser dye and in studies regarding solvation dynamics in different media (Correa and Levinger, 2006; Sánchez et al., 2018), due to the sensitivity of both absorption and emission spectra to solvent polarity and the hydrogen bond donor capacity of the solvent molecules.

Figure 2 shows that the largest C343 Stokes shifts are observed in water and PAN. This result is in agreement with their polarities and HB abilities (Correa and Levinger, 2006; Gutierrez et al., 2010). This fact is associated to the first hypothesis established for the reaction between aniline and 4-chloroquinazoline in aqueous media. Figure 2 suggests that the substrate in more acidic conditions is protonated. The high influence of PAN and water on the fluorescence spectra of C343 is related to the formation of HB, while BMIMSO does so in a lesser degree. The aforementioned response is attributed to its inability to establish HB interactions leading to a lesser red shift of the emission spectra of C343. On the other hand, BMIMDCN and BMPYRDCN in Figure 2 displays a comparable response toward the fluorescence of C343. Remember that, these RTILs were used for the hydrazine reaction. BMIMDCN and BMPYRDCN bear the same anion moiety. Therefore, the only difference toward C343 might be associated with the nature of the cation moiety. However, dicyanamide anion presents a high π density, small size and high basicity (Gazitúa et al., 2014). All these features of these RTILs enable the abstraction of an HB from hydrazine favoring the zwitterionic form for hydrazine. Note that, the *k*_*N*_ value for BMPYRDCN with respect to BMIMDCN is increased by approximately one time. However, of these two solvents BMPYRDCN is the RTIL that induces the larger Stokes shift of C343, in spite of BMIMDCN being more polar. On the other hand, BMIM^+^ cation presents significant difference in electron delocalization patterns and number of nitrogen atoms compared with BMPYR^+^ cation. Therefore, the reactivity observed suggests that the “anion effect” compensates the “cation solvent effect” (Alarcón-Espósito et al., 2016; Sánchez et al., 2018).

## Conclusions

A kinetic study on a S_N_Ar reaction in a series of reaction media and two nucleophiles of different nature have been illustrated. The reactions have been studied because the electrophile bears heteroatoms on the aromatic ring as substituent able to establish intramolecular HB that may be activated by solvation or by the nucleophile. On the other hand, the kinetic analyses shown that solvent effects are affected under a change of amine nature, showing that both nucleophiles in aqueous media are pH-dependent. However, aniline shows that the reaction rate coefficients are amplified when the acidity of the media is increased, while the inverse effect is observed with hydrazine. The kinetic study for aniline showed that is possible to postulate at first glance that prior to the nucleophilic attack, there is a protonation step that improve the reactivity of the substrate. On the other hand, the solvent effects open the possibility to establish an HB with the hydrazine moving the equilibria toward its zwitterionic form. This step would be complemented with an intramolecular HB formation that will operate as a perturbation that produces a dual response at the reaction centers by enhancing the electrophilicity of the substrate and the nucleophilicity of one of the nitrogen atom of the hydrazine molecule.

## Author contributions

BS, CC, RT, RC, and PC designed the experiments, analyzed the results, wrote, and revised the manuscript. All authors have approved the final revised manuscript. PC on behalf of The Collaborative Working Group.

### Conflict of interest statement

The authors declare that the research was conducted in the absence of any commercial or financial relationships that could be construed as a potential conflict of interest.
