# Supplementary material for: Activation of Electrophile/Nucleophile Pair by a Nucleophilic and Electrophilic Solvation in a SNAr Reaction
Source: Front Chem. 2018 Oct 23;6:509. doi: 10.3389/fchem.2018.00509 (PMC6206274; doi:10.3389/fchem.2018.00509)
Supplement: Supplementary file 1 [file Data_Sheet_1.docx]

Supplementary Material

Activation of electrophile/nucleophile pair by a nucleophilic and electrophilic solvation in a S_N_Ar reaction

**Bruno Sanchez^1^, Cristian Calderón^2^, Ricardo A. Tapia^3^, Renato Contreras^1^ and Paola R. Campodónico^2^***

**Correspondence:** Paola R. Campodónico
pcampodonico@udd.cl

**
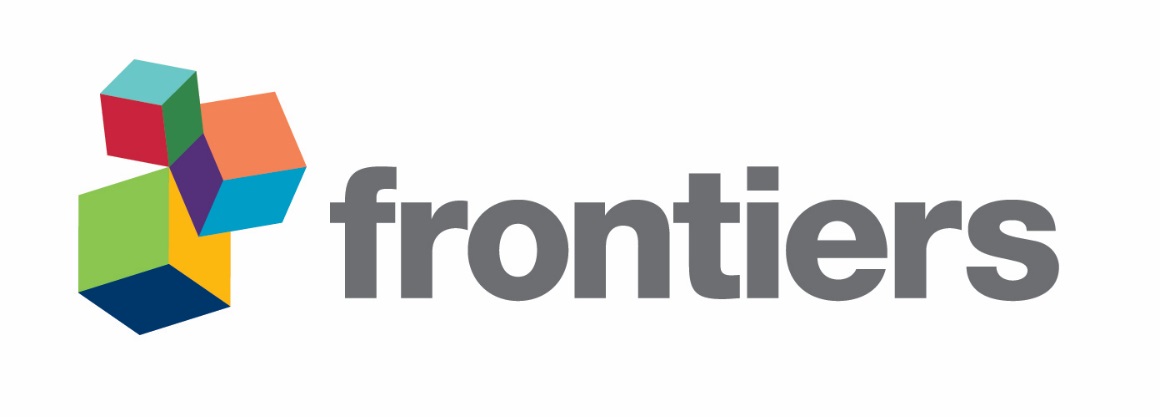
**

|  | Page |
| --- | --- |
| **Scheme S1.** Names, structures and acronyms of the ions of the ionic liquids used in the work. | 5 |
| **Table S1.** Concentration of nucleophile and observed pseudo first order rate constant for the reaction of aniline and 4-chloroquinazoline in aqueous media at a pH of 4,43±0,01 and a temperature of 25,0 ± 0,2°C. | 5 |
| **Table S2.** Concentration of nucleophile and observed pseudo first order rate constant for the reaction of aniline and 4-chloroquinazoline in aqueous media at a pH of 4,73±0,01 and a temperature of 25,0 ± 0,2°C. | 6 |
| **Table S3.** Concentration of nucleophile and observed pseudo first order rate constant for the reaction of aniline and 4-chloroquinazoline in aqueous media at a pH of 5,03±0,01 and a temperature of 25,0 ± 0,2°C. | 7 |
| **Table S4.** Concentration of nucleophile and observed pseudo first order rate constant for the reaction of hydrazine and 4-chloroquinazoline in aqueous media at a pH of 7,80±0,01 and a temperature of 25,0 ± 0,2°C. | 8 |
| **Table S5.** Concentration of nucleophile and observed pseudo first order rate constant for the reaction of hydrazine and 4-chloroquinazoline in aqueous media at a pH of 8,10±0,01 and a temperature of 25,0 ± 0,2°C. | 9 |
| **Table S6.** Concentration of nucleophile and observed pseudo first order rate constant for the reaction of hydrazine and 4-chloroquinazoline in aqueous media at a pH of 8,40±0,01 and a temperature of 25,0 ± 0,2°C. | 9 |
| **Table S7.** Observed pseudo first order rate constant and pH value of the solution for the reaction of aniline and 4-chloroquinazoline in aqueous media at a temperature of 25,0 ± 0,2°C. | 10 |
| **Table S8.** Concentration of nucleophile and observed pseudo first order rate constant for the reaction of aniline and 4-chloroquinazoline in BMIMMSO at a temperature of 25,0 ± 0,2°C. | 11 |
| **Table S9.** Concentration of nucleophile and observed pseudo first order rate constant for the reaction of aniline and 4-chloroquinazoline in EAN at a temperature of 25,0 ± 0,2°C. | 11 |
| **Table S10.** Concentration of nucleophile and observed pseudo first order rate constant for the reaction of hydrazine and 4-chloroquinazoline in ethanol at a temperature of 25,0 ± 0,2°C. | 12 |
| **Table S11.** Concentration of nucleophile and observed pseudo first order rate constant for the reaction of hydrazine and 4-chloroquinazoline in butanol at a temperature of 25,0 ± 0,2°C. | 12 |
| **Table S12.** Concentration of nucleophile and observed pseudo first order rate constant for the reaction of hydrazine and 4-chloroquinazoline in dioxane at a temperature of 25,0 ± 0,2°C. | 13 |
| **Table S13.** Concentration of nucleophile and observed pseudo first order rate constant for the reaction of hydrazine and 4-chloroquinazoline in acetonitrile at a temperature of 25,0 ± 0,2°C. | 13 |
| **Table S14.** Concentration of nucleophile and observed pseudo first order rate constant for the reaction of hydrazine and 4-chloroquinazoline in BMIMDCN at a temperature of 25,0 ± 0,2°C. | 14 |
| **Table S15.** Concentration of nucleophile and observed pseudo first order rate constant for the reaction of hydrazine and 4-chloroquinazoline in BMPYRDCN at a temperature of 25,0 ± 0,2°C. | 15 |
| **Figure S1.** Graph of pH of the solution and observed pseudo first order rate constant for the reaction of aniline and 4-chloroquinazoline. | 16 |
| **Figure S2.** Graph of the pseudo first order rate constant vs aniline concentration for the reaction of 4-chloroquinazoline and aniline on different reaction media. | 16 |
| **Figure S3.** Graph of the pseudo first order rate constant vs hydrazine concentration for the reaction of 4-chloroquinazoline and hydrazine on different conventional organic solvents. | 17 |
| **Figure S4.** Graph of the pseudo first order rate constant vs hydrazine concentration for the reaction of 4-chloroquinazoline and hydrazine on different reaction media. | 17 |
| **Scheme S2.** Reaction products for the S_N_Ar reaction between 4-chloroquinazoline and hydrazine/aniline respectively. | 18 |
| **Scheme S3.** Electrophilic and nucleophilic solvation by water molecules proposed for the studied S_N_Ar reaction. | 18 |

**
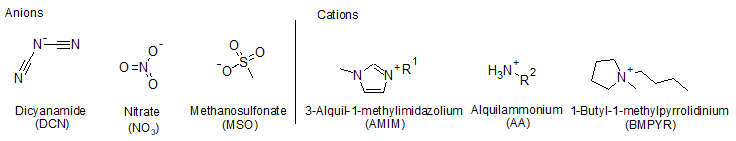
**

**Scheme S1.** Names, structures and acronyms of the ions of the ionic liquids used in the work (R1=ethyl,butyl; R2=propyl).

**Table S1.** Concentration of nucleophile and observed pseudo first order rate constant for the reaction of aniline and 4-chloroquinazoline in aqueous media at a pH of 4,43±0,01 and a temperature of 25.0 ± 0.2°C.

| Concentration of Free Aniline ∙10^3^(M) | *k_obs_* ∙10^3^ (s^-1^) |
| --- | --- |
| 0.28 | 1.52 ± 0.05 |
| 0.42 | 3.10 ± 0.09 |
| 0.56 | 4.83 ± 0.10 |
| 0.70 | 5.56 ± 0.12 |
| 0.84 | 7.45 ± 0.15 |
| 1.12 | 9.77 ± 0.12 |
| 1.40 | 12.60 ± 0.20 |
| 1.68 | 15.20 ± 0.17 |
| 1,95 | 20.60 ± 0.34 |

**Table S2.** Concentration of nucleophile and observed pseudo first order rate constant for the reaction of aniline and 4-chloroquinazoline in aqueous media at a pH of 4.73±0.01 and a temperature of 25.0 ± 0.2°C.

| Concentration of Free Aniline ∙10^3^ (M) | *k_obs_* ∙10^3^ (s^-1^) |
| --- | --- |
| 0.42 | 1.36 ± 0.09 |
| 0.85 | 2.63 ± 0.08 |
| 1.06 | 3.79 ± 0.09 |
| 1.27 | 5.04 ± 0.09 |
| 1.48 | 5.41 ± 0.08 |
| 1.69 | 6.59 ± 0.15 |
| 1.90 | 7.09 ± 0.07 |
| 2.11 | 7.79 ± 0.012 |

**Table S3.** Concentration of nucleophile and observed pseudo first order rate constant for the reaction of aniline and 4-chloroquinazoline in aqueous media at a pH of 5.03±0.01 and a temperature of 25.0 ± 0.2°C.

| Concentration of Free Aniline ∙10^3^(M) | *k_obs_* ∙10^3^ (s^-1^) |
| --- | --- |
| 0.57 | 0.80 ± 0.03 |
| 0.86 | 1.58 ± 0.05 |
| 1.14 | 1.92 ± 0.07 |
| 1.43 | 2.27 ± 0.06 |
| 1.71 | 3.10 ± 0.08 |
| 2.00 | 2.83 ± 0.07 |
| 2.28 | 3.45 ± 0.09 |
| 2.57 | 4.14 ± 0.06 |
| 2.85 | 4.94 ± 0.11 |
| 3.42 | 6.00 ± 0.11 |
| 3.71 | 6.10 ± 0.09 |
| 3.99 | 7.30 ± 0.15 |

**Table S4.** Concentration of nucleophile and observed pseudo first order rate constant for the reaction of hydrazine and 4-chloroquinazoline in aqueous media at a pH of 7.80±0.01 and a temperature of 25.0 ± 0.2°C.

| Concentration of Free Hydrazine ∙10^2^(M) | *k_obs_* ∙10^3^ (s^-1^) |
| --- | --- |
| 6.08 | 1.43 ± 0.05 |
| 9.13 | 2.36 ± 0.05 |
| 12.17 | 3.35 ± 0.16 |
| 15.21 | 4.09 ± 0.13 |
| 18.25 | 4.67 ± 0.18 |
| 21.29 | 6.03 ± 0.20 |
| 24.34 | 6.83 ± 0.21 |

**Table S5.** Concentration of nucleophile and observed pseudo first order rate constant for the reaction of hydrazine and 4-chloroquinazoline in aqueous media at a pH of 8.10±0.01 and a temperature of 25.0 ± 0.2°C.

| Concentration of Free Hydrazine ∙10^2^(M) | *k_obs_* ∙10^3^ (s^-1^) |
| --- | --- |
| 4.40 | 2.68 ± 0.12 |
| 6.02 | 3.34 ± 0.14 |
| 8.02 | 4.36 ± 0.16 |
| 11.11 | 5.36 ± 0.25 |
| 15.05 | 7.21 ± 0.21 |
| 17.05 | 8.83 ± 0.46 |
| 19.06 | 9.77 ± 0.22 |

**Table S6.** Concentration of nucleophile and observed pseudo first order rate constant for the reaction of hydrazine and 4-chloroquinazoline in aqueous media at a pH of 8.40±0.01 and a temperature of 25.0 ± 0.2°C.

| Concentration of Free Hydrazine ∙10^2^(M) | *k_obs_* ∙10^3^ (s^-1^) |
| --- | --- |
| 5.97 | 3.78 ± 0.09 |
| 7.46 | 4.62 ± 0.14 |
| 8.95 | 6.08 ± 0.15 |
| 13.43 | 8.41 ± 0.21 |
| 16.41 | 10.97 ± 0.27 |
| 19.40 | 12.66 ± 0.38 |
| 22.38 | 14.44 ± 0.32 |

**Table S7.** Observed pseudo first order rate constant and pH value of the solution for the reaction of aniline and 4-chloroquinazoline in aqueous media at a temperature of 25.0 ± 0.2°C.

| pH | *k_obs_* ∙10^3^ (s^-1^) |
| --- | --- |
| 1.00 | 1.505 ± 0.026 |
| 1.85 | 1.273 ± 0.028 |
| 2.50 | 1.297 ± 0.041 |
| 3.03 | 1.122 ± 0.032 |
| 3.51 | 1.068 ± 0.031 |
| 3.70 | 1.034 ± 0.026 |
| 4.02 | 0.936 ± 0.029 |
| 4.20 | 0.865 ± 0.031 |
| 4.50 | 0.819 ± 0.025 |
| 4.70 | 0.654 ± 0.023 |
| 5.07 | 0.415 ± 0.017 |
| 5.20 | 0.419 ± 0.016 |
| 5.55 | 0.225 ± 0.011 |
| 6.00 | 0.091 ± 0.004 |
| 6.20 | 0.060 ± 0.003 |
| 6.48 | 0.031 ± 0.003 |
| 7.00 | 0.022 ± 0.002 |
| 7.50 | 0.020 ± 0.002 |
| 8.00 | 0.019 ± 0.002 |

**Table S8.** Concentration of nucleophile and observed pseudo first order rate constant for the reaction of aniline and 4-chloroquinazoline in BMIMMSO at a temperature of 25.0 ± 0.2°C.

| Concentration of Aniline ∙10^3^(M) | *k_obs_* ∙10^3^ (s^-1^) |
| --- | --- |
| 4.92 | 0.38 ± 0.08 |
| 8.20 | 0.41 ± 0.05 |
| 11.48 | 0.45 ± 0.06 |
| 14.76 | 0.46 ± 0.07 |
| 18.04 | 0.55 ± 0.09 |
| 24.60 | 0.61 ± 0.10 |
| 27.88 | 0.65 ± 0.11 |

**Table S9.** Concentration of nucleophile and observed pseudo first order rate constant for the reaction of aniline and 4-chloroquinazoline in PAN at a temperature of 25.0 ± 0.2°C.

| Concentration of Aniline ∙10^3^(M) | *k_obs_* ∙10^3^ (s^-1^) |
| --- | --- |
| 3.05 | 15.1 ± 0.42 |
| 4.71 | 17.22 ± 0.56 |
| 7.85 | 19.27 ± 0.53 |
| 11.10 | 22.05 ± 0.21 |
| 14.13 | 25.17 ± 0.41 |
| 17.27 | 27.99 ± 0.28 |
|  |  |

**Table S10.** Concentration of nucleophile and observed pseudo first order rate constant for the reaction of hydrazine and 4-chloroquinazoline in ethanol at a temperature of 25.0 ± 0.2°C.

| Concentration of Free Hydrazine ∙10^2^(M) | *k_obs_* ∙10^3^ (s^-1^) |
| --- | --- |
| 1.50 | 2.21 **±** 0.31 |
| 2.00 | 3.18 **±** 0.07 |
| 2.50 | 3.97 **±** 0.19 |
| 3.00 | 4.31 **±** 0.08 |
| 3.50 | 4.85 **±** 0.07 |
| 4.00 | 6.26 **±** 0.26 |
| 4.50 | 6.46 **±** 0.10 |

**Table S11.** Concentration of nucleophile and observed pseudo first order rate constant for the reaction of hydrazine and 4-chloroquinazoline in butanol at a temperature of 25.0 ± 0.2°C.

| Concentration of Free Hydrazine ∙10^2^(M) | *k_obs_* ∙10^3^ (s^-1^) |
| --- | --- |
| 0.69 | 1.10 **±** 0.10 |
| 1.38 | 2.03 **±** 0.09 |
| 3.45 | 4.74 **±** 0.17 |
| 4.83 | 7.33 **±** 0.12 |
| 6.21 | 9.22 **±** 0.08 |
| 6.90 | 8.99 **±** 0.45 |

**Table S12.** Concentration of nucleophile and observed pseudo first order rate constant for the reaction of hydrazine and 4-chloroquinazoline in dioxane at a temperature of 25.0 ± 0.2°C.

| Concentration of Free Hydrazine ∙10^2^(M) | *k_obs_* ∙10^3^ (s^-1^) |
| --- | --- |
| 3.81 | 1.72 **±** 0.05 |
| 4.35 | 1.75 **±** 0.05 |
| 4.90 | 2.21 **±** 0.06 |
| 5.44 | 2.61 **±** 0.12 |
| 6.53 | 2.99 **±** 0.23 |
| 7.62 | 3.65 **±** 0.07 |
| 8.71 | 4.23 **±** 0.20 |

**Table S13.** Concentration of nucleophile and observed pseudo first order rate constant for the reaction of hydrazine and 4-chloroquinazoline in acetonitrile at a temperature of 25.0 ± 0.2°C.

| Concentration of Free Hydrazine ∙10^2^(M) | *k_obs_* ∙10^3^ (s^-1^) |
| --- | --- |
| 3.27 | 1.25 **±** 0.07 |
| 4.09 | 1.38 **±**0.07 |
| 4.60 | 1.54 **±** 0.08 |
| 5.11 | 1.74 **±** 0.08 |
| 6.13 | 1.98 **±** 0.04 |
| 7.15 | 2.04 **±** 0.04 |
| 8.18 | 2.41 **±** 0.04 |

**Table S14.** Concentration of nucleophile and observed pseudo first order rate constant for the reaction of hydrazine and 4-chloroquinazoline in BMIMDCN at a temperature of 25.0 ± 0.2°C.

| Concentration of Free Hydrazine ∙10^2^(M) | *k_obs_* ∙10^3^ (s^-1^) |
| --- | --- |
| 0.89 | 2.50 **±** 0.50 |
| 1.34 | 4.89 **±** 0.62 |
| 1.78 | 6.49 **±** 0.47 |
| 2.23 | 9.65 **±** 0.60 |
| 2.48 | 10.45 **±** 1.07 |
| 2.68 | 11.72 **±** 1.10 |
| 3.12 | 12.63 **±** 1.19 |

**Table S15.** Concentration of nucleophile and observed pseudo first order rate constant for the reaction of hydrazine and 4-chloroquinazoline in BMPYRDCN at a temperature of 25.0 ± 0.2°C.

| Concentration of Free Hydrazine ∙10^2^(M) | *k_obs_* ∙10^3^ (s^-1^) |
| --- | --- |
| 0.38 | 2.42 ± 0.38 |
| 0.75 | 4.82 ± 0.88 |
| 1.13 | 7.60 ± 0.90 |
| 1.50 | 8.27 ± 0.77 |
| 1.88 | 12.99 ± 1.02 |
| 2.25 | 14.79 ± 0.95 |
| 2.63 | 16.75 ± 1.52 |

**Figure S1.** Graph of pH of the solution and observed pseudo first order rate constant for the reaction of aniline and 4-chloroquinazoline.


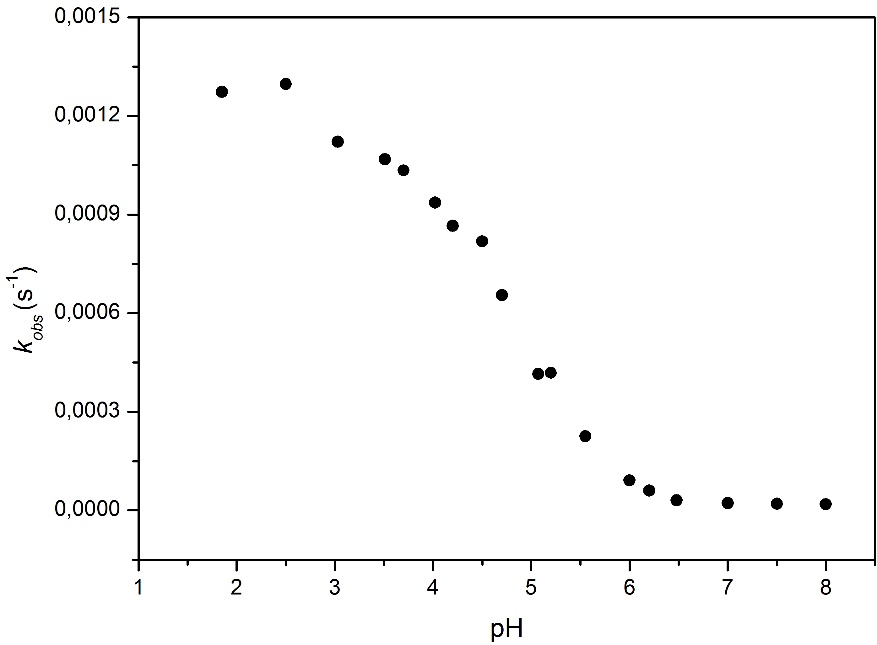


**Figure S2.** Graph of the pseudo first order rate constant vs aniline concentration for the reaction of 4-chloroquinazoline and aniline on different reaction media.

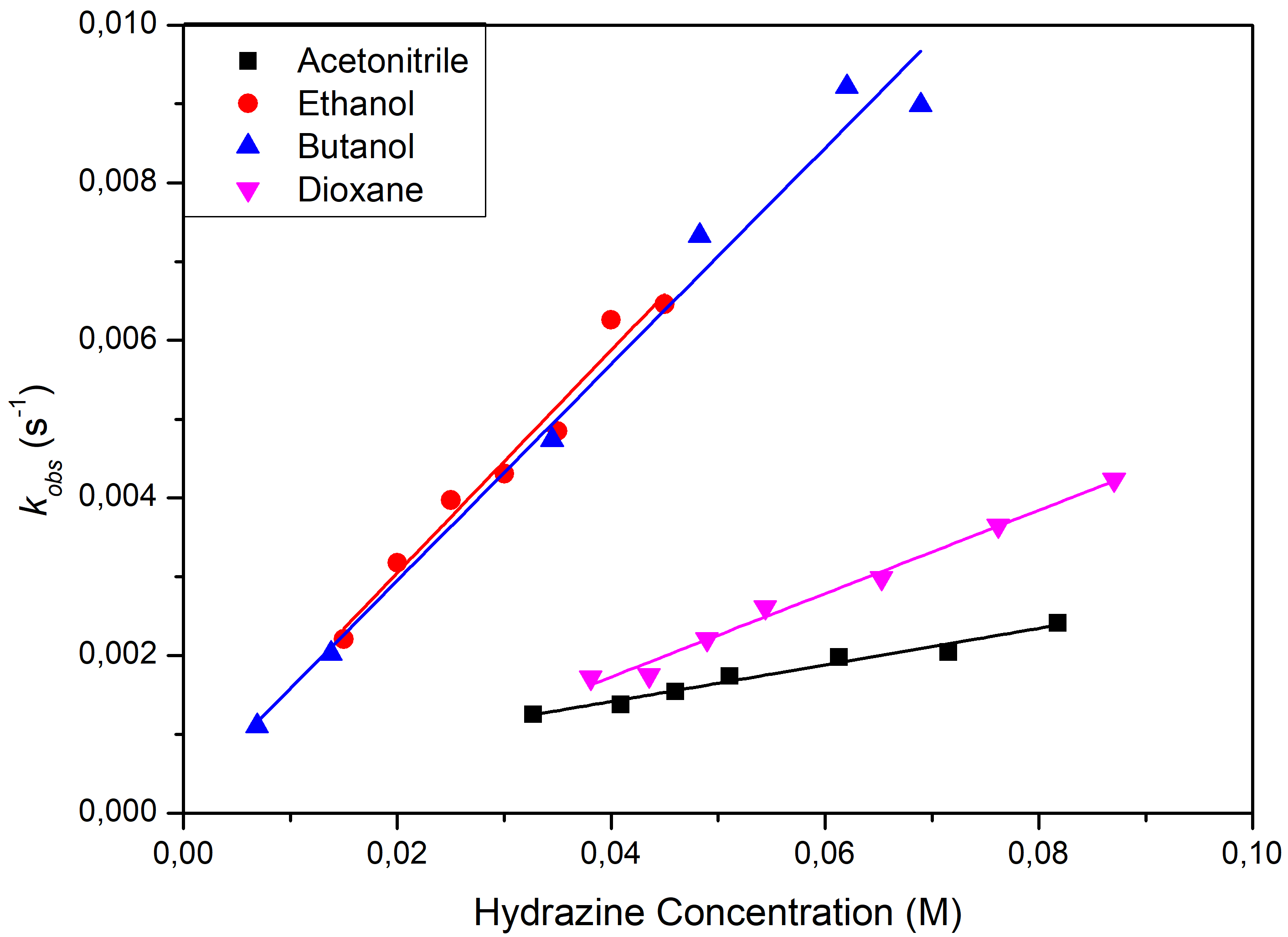
**Figure S3.** Graph of the pseudo first order rate constant vs hydrazine concentration for the reaction of 4-chloroquinazoline and hydrazine on different conventional organic solvents.


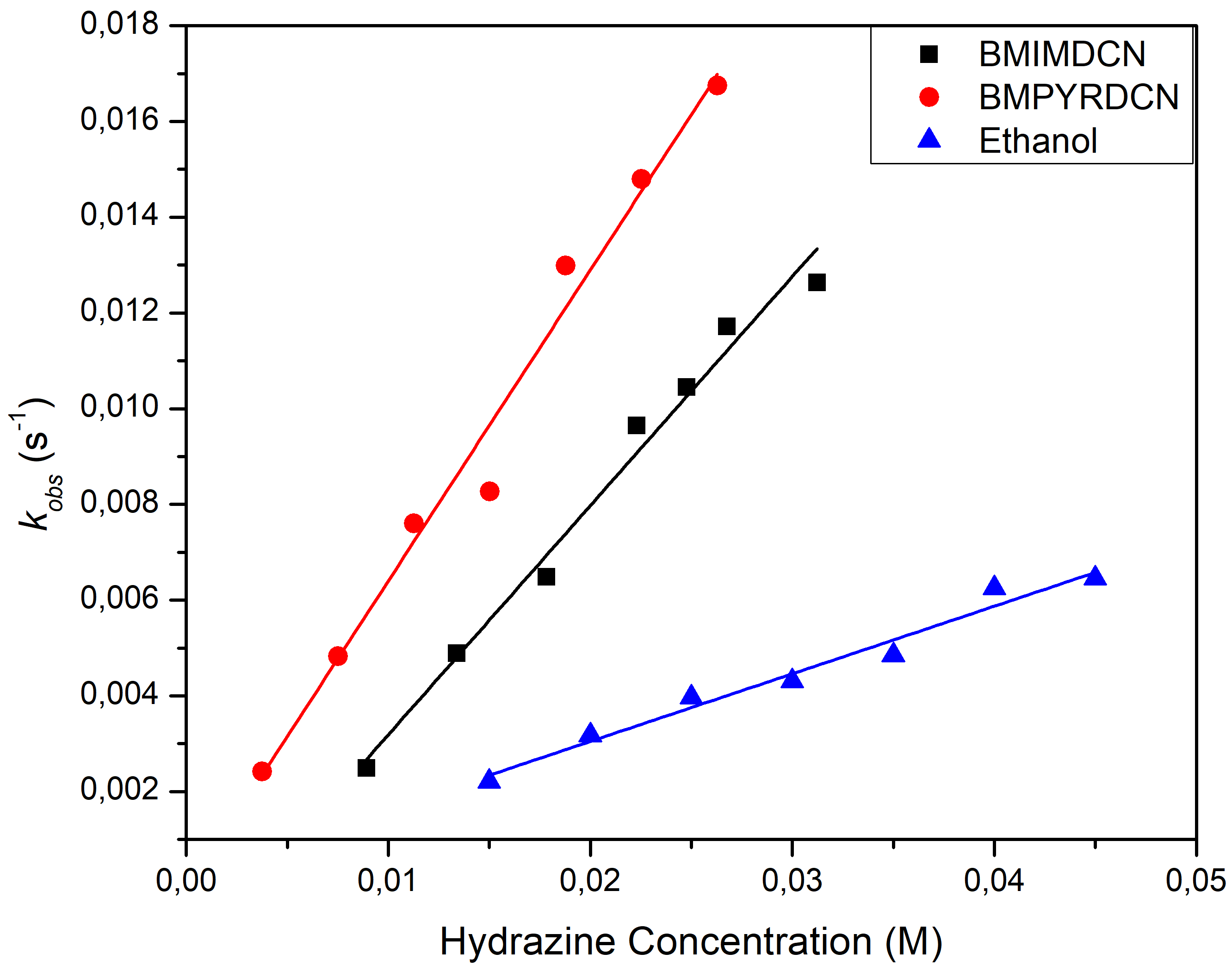
**Figure S4.** Graph of the pseudo first order rate constant vs hydrazine concentration for the reaction of 4-chloroquinazoline and hydrazine on different reaction media.





**Scheme S3.** Reaction products for the S_N_Ar reaction between 4-chloroquinazoline and hydrazine/aniline, respectively.





**Scheme S3.** Electrophilic and nucleophilic solvation by water molecules proposed for the studied S_N_Ar reaction.
